# Carcinosarcoma of Vater’s papilla: case report of a rare neoplasm and review of the literature

**DOI:** 10.1186/s40792-019-0575-z

**Published:** 2019-01-31

**Authors:** Rumi Itoyama, Yo-ichi Yamashita, Yosuke Nakao, Toshihiko Yusa, Naoki Umezaki, Takanobu Yamao, Shigeki Nakagawa, Hirohisa Okabe, Katsunori Imai, Hiromitsu Hayashi, Daisuke Hashimoto, Akira Chikamoto, Hideo Baba

**Affiliations:** 1 Department of Gastroenterological Surgery, Kumamoto University Graduate School of Medical Sciences, 1-1-1 Honjo, Kumamoto, 860-8556 Japan; 2 Department of Gastroenterological Surgery, Omuta Tenryo Hospital, 1-100 Tenryo, Omuta, 836-8566 Japan

**Keywords:** Carcinosarcoma, Vater’s papilla, Stomach-preserving pancreaticoduodenectomy, Chemotherapy

## Abstract

**Background:**

Carcinosarcoma is a rare tumor that includes both carcinoma and sarcoma components. It develops commonly in the female reproductive tract, most often in the uterus. However, as there are a small number of similar cases in the English literature, we would like to present a rare case of a carcinosarcoma in Vater’s papilla.

**Case presentation:**

A 76-year-old female patient was preoperatively diagnosed with a papillary adenocarcinoma in Vater’s papilla by endoscopic biopsy. The patient underwent subtotal stomach-preserving pancreaticoduodenectomy, and postoperative pathological examination diagnosed the carcinosarcoma. The patient received adjuvant chemotherapy with gemcitabine, but multiple liver metastases were found 3 months after the operation. Though chemotherapy with gemcitabine and cisplatin was introduced, she died owing to tumor progression 7 months after the operation.

**Conclusion:**

Because carcinosarcoma of Vater’s papilla is a rare disease, a suitable treatment strategy has been unclear. We also present a review of the English literature regarding carcinosarcoma of Vater’s papilla.

## Background

Carcinosarcoma is a rare tumor that includes both carcinomatous and sarcomatous components [1, 2]. It occurs in the uterus most often and has been reported in lung and breast [1]. It also infrequently develops in gastrointestinal organs such as the liver, bile duct, pancreas, and intestine [3, 4]. Almost all reported cases have been primarily treated by surgical therapy [3, 5]. Even if the surgery achieves curative resection, the prognosis of carcinosarcoma is generally poor [3, 6]. As there were small numbers of similar cases in the English literature, we would like to present a rare case of carcinosarcoma of Vater’s papilla. The histological type of a malignant tumor in Vater’s papilla is usually adenocarcinoma, and its prognosis is usually good [7–9]. However, our reported case showed poor prognosis. We also present a review of the English literature regarding cases of carcinosarcoma that developed in Vater’s papilla.

## Case presentation

**Fig. 1:**
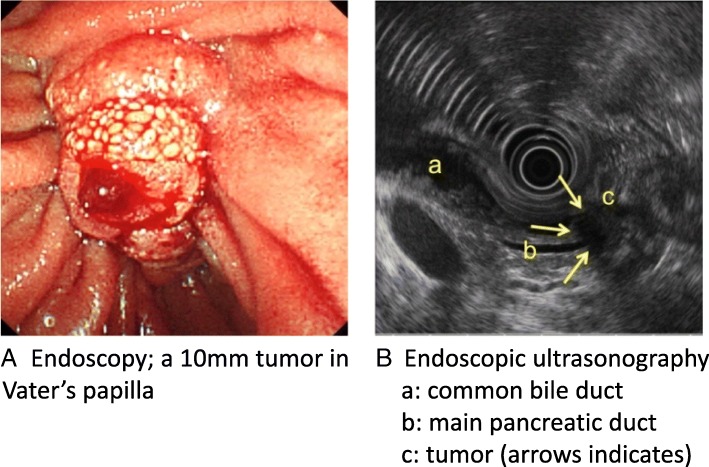
Preoperative endoscopy. Endoscopy revealed a 10-mm tumor in Vater’s papilla and endoscopic retrograde biliary drainage was placed (**A**). Endoscopic ultrasonography revealed that the tumor (arrows) invaded pancreatic parenchyma (**B**). a, common bile duct; b, main pancreatic duct; c, duodenum

**Fig. 2:**
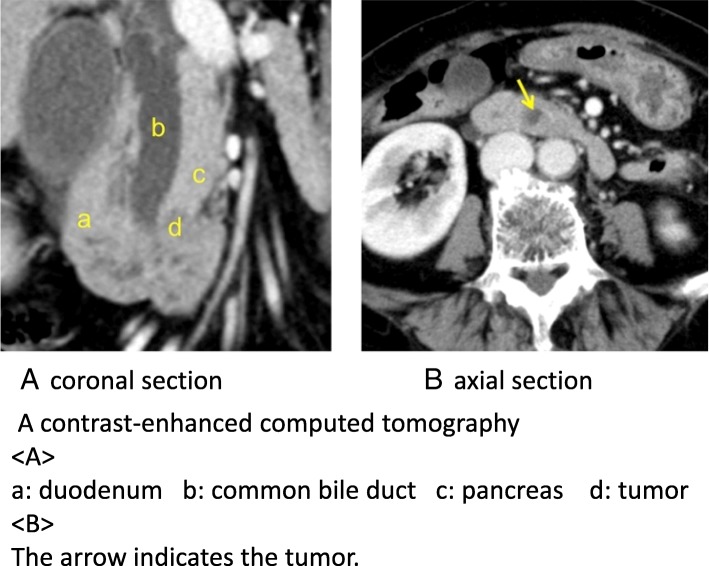
Preoperative computed tomography. Coronal section (**A**) and axial section (**B**) of a contrast-enhanced computed tomography scan revealed a hypovascular tumor (arrows) at Vater’s papilla had invaded into the pancreas directly. a, duodenum; b, common bile duct; c, pancreas; d, tumor

A 76-year-old-woman was evaluated because of general fatigue, loss of appetite, and jaundice. Laboratory test showed an elevation of total bilirubin (7.7 mg/dL) and hepatobiliary enzyme. An endoscopy showed a 10-mm tumor in Vater’s papilla (Fig. 1A), and endoscopic retrograde biliary drainage (ERBD) was placed for obstructive jaundice. After that, total bilirubin was decreased to 1.9 mg/dL. The pathological diagnosis of endoscopic biopsy of the tumor was a papillary adenocarcinoma. Endoscopic ultrasonography (EUS) revealed that this tumor invaded pancreatic parenchyma (Fig. 1B). A contrast-enhanced computed tomography (CT) revealed a hypovascular mass at Vater’s papilla (Fig. 2A, B). No evidence of distant metastasis was identified. Carcinoembryonic antigen (CEA) and carbohydrate antigen 19-9 (CA19-9) were not elevated (CEA 1.9 ng/ml, CA19-9 31.5 U/ml). Thus, the patient was preoperatively diagnosed with an adenocarcinoma of Vater’s papilla and underwent an operation.

A subtotal stomach-preserving pancreaticoduodenectomy (SSPPD) with D2 lymph node dissection was performed. The pancreas was soft and non-fibrotic. The operation time was 6 h 18 min, and the intraoperative blood loss was 417 g.

The patient developed postoperative pancreatic fistula (grade B) in accordance with the International Study Group for Pancreatic Fistula definition [10]. Appropriate persistent drainage was performed, and the patient recovered immediately and was discharged on the 30th postoperative day.

**Fig. 3:**
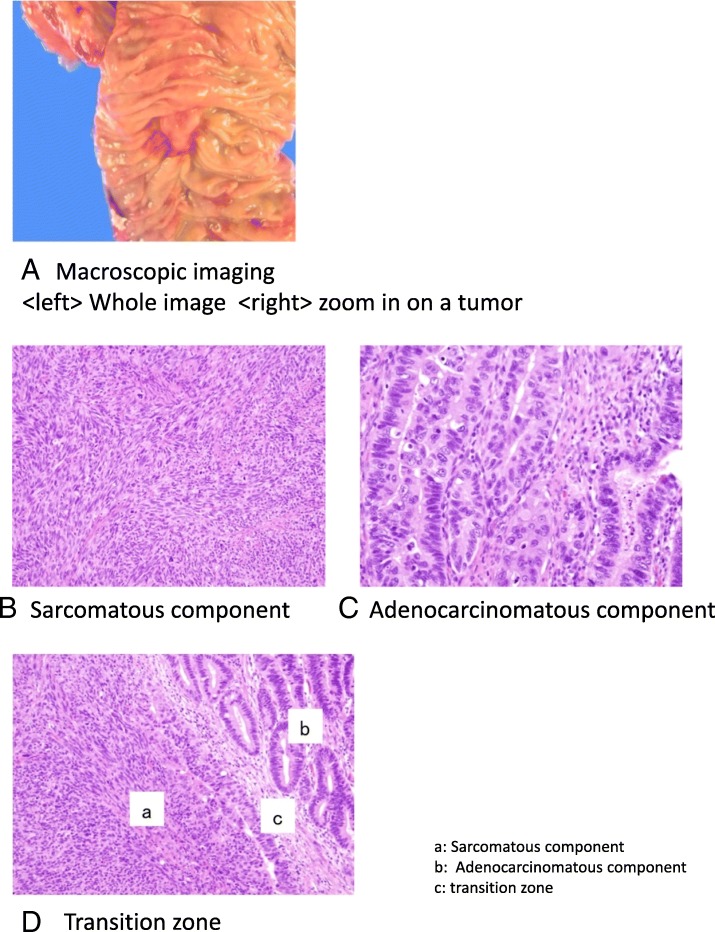
Pathological findings. Macroscopically, a 2.0 × 1.4 cm elastic hard tumor was found at Vater’s papilla (**A**). The microscopic examination of the specimen showed that spindle cells, which constructed sarcomatous tissue proliferated with intricate infiltration (**B**) and growth of tubular adenocarcinoma (**C**). Two components existed across the transition zone (**D**). a, sarcomatous component; b, adenocarcinomatous component; c, transition zone

Macroscopically, a 2.0 × 1.4 cm elastic hard tumor was found at Vater’s papilla (Fig. 3A). The microscopic examination of the specimen showed that spindle cells that constructed sarcomatous tissue proliferated with intricate infiltration (Fig. 3B) and growth of tubular adenocarcinoma (Fig. 3C). Two components existed across the transition zone (Fig. 3D). Approximately 30% of the tumor was sarcoma component, and the remainder was carcinoma component. The tumor directly infiltrated into peripancreatic fatty tissue, pancreatic parenchyma, and a lymph node. Finally, pathological diagnosis was carcinosarcoma of Vater’s papilla. Resection margins were pathologically negative; thus, curative resection was achieved.

The patient received adjuvant chemotherapy using gemcitabine 1 month after the operation. It was continued without any obvious adverse events; however, an enhanced CT revealed multiple liver metastases at 3 months after the operation. The chemotherapy was changed to gemcitabine plus cisplatin. However, enhanced CT revealed the rapid progression of the metastasis at 6 months after the operation. The patient died at 7 months after the operation due to the continuous tumor progression.

## Discussion

Carcinosarcoma is defined as a malignant neoplasm with both epithelial and mesenchymal elements within the same tissue [1]. It is a rare tumor, comprising less than 1% of all malignant neoplasms of the hepatobiliary tract. Carcinosarcoma of Vater’s papilla is extremely rare [3]. We found only five cases (excluding our current case) among current English literatures (Table 1) [7–9, 11, 12]. In all cases, patients had some subjective symptoms, such as jaundice, loss of appetite, and malaise. All patients underwent pancreaticoduodenectomy. Generally, their prognosis of carcinosarcoma of the hepatobiliary tract including Vater’s papilla is poor, as the overall 1-, 3-, and 5-year survival rates for patients with carcinosarcoma of the hepatobiliary tract after surgery are 44.0%, 29.3%, and 27.0%, respectively [3].

**Table 1:** Previous reports about cases of carcinosarcoma of Vater’s papilla

Author	Year	Sex	Age	Chief complaint	Operation	Adjuvant therapy	Lymph node metastasis	Prognosis
Kench [11]	1997	F	46	Melena, fatigue, dyspnea	PD	NA	Positive	Died (8 POM)
Kijima [12]	1999	M	46	Jaundice, liver dysfunction	PD	NA	NA	NA
Tanaka [7]	2008	F	70	Jaundice, loss of appetite	PPPD	NA	Positive	Alive (24 POM)
Rao [8]	2016	M	67	Abdominal discomfort, weight loss	PD	None	Positive	Alive (5 POM)
Izumi [9]	2016	M	73	Jaundice	SSPPD	None	Negative	Alive (5 POM)
This case		F	76	Jaundice, loss of appetite, general malaise	SSPPD	Gemcitabine	Positive	Died (7 POM)

*NA* not available, *PD* pancreaticoduodenectomy, *POM* postoperative month, *PPPD* pylorus-preserving pancreatoduodenectomy, *SSPPD* substomach-preserving pancreatoduodenectomy

Preoperative diagnose of carcinosarcoma is difficult. Recently, several studies showed that 18F-fluorodeoxyglucose(FDG) positron emission tomography-computed tomography (PET-CT) was useful for diagnosing of carcinosarcoma, because it showed intense FDG uptake in a patient with carcinosarcoma [13–15]. In our case, preoperative PET-CT was not performed. If it showed abnormally intense FDG uptake despite being a small tumor, then it may have become an evidence for judging before the operation. However, high SUVmax value of the tumor does not influence the treatment strategy. So, in fact, it is not realistic to perform PET-CT for all patients with tumor of Vater’s papilla.

Only our case received adjuvant chemotherapy using gemcitabine. However, our case developed liver metastasis 3 months after the operation. Thus, the effect of adjuvant chemotherapy and a suitable regimen remains unclear. Because it is a rare tumor, also, the suitable treatment of metastatic carcinosarcoma of the biliary tract, including Vater’s papilla, has not been established. Our patient received gemcitabine plus cisplatin for multiple liver metastases, following treatment strategy for metastatic biliary duct cancer. However, this regimen was not effective in our case. In the gynecological field, there are some reports that chemotherapy using ifosfamide, cisplatin, paclitaxel, or carboplatin are effective for carcinosarcoma [16, 17]. And in the respiratory division, there is a report that nab-paclitaxel plus carboplatin is effective and safe for pulmonary carcinosarcoma [18]. Combination chemotherapy that is effective for both carcinoma and sarcoma might be considered for carcinosarcoma [19].

There were some reports that cancer metastasis or recurrence revealed along the catheter tract of biliary drainage [20], or the patients with ampullary cancer who had preoperative biliary drainage, had poor prognosis [21]. Recently, a report was published that patients who underwent preoperative endoscopic retrograde cholangiopancreatography (ERCP) had a significantly higher rate of early distant metastasis within 1 year, especially in patients with early-stage cancer of Vater’s papilla [22]. And we also think there is a possibility that preoperative biliary drainage may be one of the possible reasons why this patient had an early recurrence. ERBD was placed for obstructive jaundice in this patient. So, it is necessary to keep it in mind that these invasive procedures may cause disruption or dissemination of cancer cells.

## Conclusion

Because carcinosarcoma of Vater’s papilla is a rare disease, a suitable treatment strategy has been unclear. Curative resection may contribute to a better prognosis; however, adjuvant chemotherapy and treatment for metastatic disease should be discussed more in the future.

## Abbreviations

CA19-9: Carbohydrate antigen 19-9
CEA: Carcinoembryonic antigen
CT: Contrast-enhanced computed tomography
ERBD: Endoscopic retrograde biliary drainage
ERCP: Endoscopic retrograde cholangiopancreatography
EUS: Endoscopic ultrasonography
FDG: Fluorodeoxyglucose
PET-CT: Positron emission tomography-computed tomography
SSPPD: Subtotal stomach-preserving pancreaticoduodenectomy

## References

[1] Guy JB, Trone JC, Casteillo F, Forest F, Pacaut C, Moncharmont C, Espenel S, Vallard A, Langrand Escure J, Collard O (2014). Carcinosarcomas in female genital tracts: general review. Bull Cancer.

[2] Anupama R, Kuriakose S, Vijaykumar DK, Pavithran K, Jojo A, Indu RN, Sheejamol VS (2013). Carcinosarcoma of the uterus-a single institution retrospective analysis of the management and outcome and a brief review of literature. Indian J Surg Oncol.

[3] Okabayashi T, Shima Y, Iwata J, Iiyama T, Sumiyoshi T, Kozuki A, Tokumaru T, Hata Y, Noda Y, Morita M (2014). Surgical outcomes for 131 cases of carcinosarcoma of the hepatobiliary tract. J Gastroenterol.

[4] Iida T, Kaneto H, Ishigami K, Naito T, Nakagaki S, Satoh S, Shimizu H, Sasaki K, Konishi Y, Kon S (2014). A case of carcinosarcoma of the duodenum. Nihon Shokakibyo Gakkai Zasshi.

[5] Benoit L, Arnould L, Cheynel N, Goui S, Collin F, Fraisse J, Cuisenier J (2005). The role of surgery and treatment trends in uterine sarcoma. Eur J Surg Oncol.

[6] Singh R (2014). Review literature on uterine carcinosarcoma. J Cancer Res Ther.

[7] Tanaka A, Hirabayashi K, Tobita K, Okamura T, Takashimizu S, Ishii M, Dowaki S, Yasuda M, Mine T, Ogoshi K (2008). Carcinosarcoma of the ampulla of Vater. J Clin Gastroenterol.

[8] Rao P, Sikora SS, Narayanaswamy S, Ghosal N, Kini D (2016). Ampullary carcinosarcoma with osteosarcomatous, small cell neuroendocrine carcinoma and conventional adenocarcinoma components; first report. Pathol Res Pract.

[9] Izumi H, Yazawa N, Furukawa D, Masuoka Y, Yamada M, Mashiko T, Kawashima Y, Ogawa M, Kawaguchi Y, Mine T (2016). Carcinosarcoma of the ampulla of Vater: a case report and literature review. Surg Case Rep.

[10] Bassi C, Marchegiani G, Dervenis C, Sarr M, Abu Hilal M, Adham M, Allen P, Andersson R, Asbun HJ, Besselink MG (2017). The 2016 update of the International Study Group (ISGPS) definition and grading of postoperative pancreatic fistula: 11 years after. Surgery.

[11] Kench JG, Frommer DJ (1997). Sarcomatoid carcinoma of the ampulla of Vater. Pathology.

[12] Kijima H, Takeshita T, Suzuki H, Tanahashi T, Suto A, Izumika H, Miki H, Terasaki Y, Nakamura M, Watanabe H (1999). Carcinosarcoma of the ampulla of Vater: a case report with immunohistochemical and ultrastructural studies. Am J Gastroenterol.

[13] Chuang TL, Lai CL, Chang SM, Wang YF (2012). Pulmonary carcinosarcoma: 18F-FDG PET/CT imaging. Clin Nucl Med.

[14] Li B, Zhang Y, Hou J, Yu H, Shi H (2016). Primary liver Carcinosarcoma and 18F-FDG PET/CT. Clin Nucl Med.

[15] Dong A, Dong H, Jing W, Zuo C (2016). FDG PET/CT in sarcomatoid carcinoma of the gallbladder with chondroid differentiation. Clin Nucl Med.

[16] Dandamudi RK, Aslam S, Walji N, El-Modir A, Fernando I (2015). Chemotherapy for uterine carcinosarcoma with carboplatin, ifosfamide and mesna. Anticancer Res.

[17] Brackmann M, Stasenko M, Uppal S, Erba J, Reynolds RK, McLean K (2018). Comparison of first-line chemotherapy regimens for ovarian carcinosarcoma: a single institution case series and review of the literature. BMC Cancer.

[18] Niwa H, Otani S, Nakada N, Sasaki J, Saka H, Masuda N (2018). Nab-paclitaxel plus carboplatin as an effective and safe chemotherapy regimen for pulmonary carcinosarcoma with interstitial lung disease: a case report. Respir Med Case Rep.

[19] Miyakawa A, Ishibashi H, Takashina A, Ishigaki K, Iwai Y, Abe H, Katagiri T, Sakaguchi Y, Matsushima T, Tamura T (2014). A case of gastric carcinosarcoma treated with surgical resection and irinotecan and mitomycin C combination therapy. Nihon Shokakibyo Gakkai Zasshi.

[20] Patel D, Lu Y (2015). FDG-PET/CT manifestation of tumor seeding along percutaneous biliary drainage catheter tract. Clin Nucl Med.

[21] Barauskas G, Urbonas K, Smailyte G, Pranys D, Pundzius J, Gulbinas A (2016). Preoperative endoscopic biliary drainage may negatively impact survival following pancreatoduodenectomy for ampullary cancer. Dig Surg.

[22] Ahn KS, Kang KJ, Kim YH, Lee YS, Cho GB, Kim TS, Lee JW (2018). Impact of preoperative endoscopic cholangiography and biliary drainage in ampulla of Vater cancer. Surg Oncol.

